# Molecular mechanisms of metabolic associated fatty liver disease (MAFLD): functional analysis of lipid metabolism pathways

**DOI:** 10.1042/CS20220572

**Published:** 2022-09-23

**Authors:** Olufunto O. Badmus, Sarah A. Hillhouse, Christopher D. Anderson, Terry D. Hinds, David E. Stec

**Affiliations:** 1 Department of Physiology and Biophysics, Cardiorenal, and Metabolic Diseases Research Center, University of Mississippi Medical Center, Jackson, MS 39216, U.S.A.; 2 Department of Surgery, University of Mississippi Medical Center, Jackson, MS 39216, U.S.A.; 3 Department of Pharmacology and Nutritional Sciences, Barnstable Brown Diabetes Center, Markey Cancer Center, University of Kentucky, Lexington, KY 40508, U.S.A.

**Keywords:** hepatic steatosis, intracellular signaling, non alcoholic fatty liver disease, obesity

## Abstract

The metabolic-associated fatty liver disease (MAFLD) is a condition of fat accumulation in the liver in combination with metabolic dysfunction in the form of overweight or obesity and insulin resistance. It is also associated with an increased cardiovascular disease risk, including hypertension and atherosclerosis. Hepatic lipid metabolism is regulated by a combination of the uptake and export of fatty acids, *de novo* lipogenesis, and fat utilization by β-oxidation. When the balance between these pathways is altered, hepatic lipid accumulation commences, and long-term activation of inflammatory and fibrotic pathways can progress to worsen the liver disease. This review discusses the details of the molecular mechanisms regulating hepatic lipids and the emerging therapies targeting these pathways as potential future treatments for MAFLD.

## Introduction

The levels of obesity are at an all-time high, and this has led to an increase in obesity-associated comorbidities such as insulin-resistant Type 2 diabetes mellitus (T2DM), fatty liver, and cardiovascular diseases. The accumulation of fat in the liver, or simple steatosis, is a benign condition that is a precursor to events that can progress to non-alcoholic fatty liver disease (NAFLD), which is one of the most common causes of chronic liver disease. NAFLD has a global prevalence estimated at 25% [1,2], and its occurrence is increasing in developed and underdeveloped countries [3]. Within the United States, the prevalence is estimated at 24%, with a projected increase of 17.8 million cases from 2015 to 2030 [1,4,5]. In NAFLD, hepatic lipid accumulation is associated with metabolic and cardiovascular diseases like obesity, T2DM, hypertriglyceridemia, and hypertension [6]. Due to this fact, a name change to the metabolic (dysfunction) associated fatty liver disease (MAFLD) has been proposed [2,7]. Patients with MAFLD commonly develop cardiovascular diseases such as hypertension, atherosclerosis, and heart and kidney diseases. For example, 80–90% of obese and 70–80% of T2DM patients typically have MAFLD [8,9]. However, this finding is not universal as others have not revealed an increased risk of cardiovascular disease in MAFLD patients, likely due to the confounding effects of social class [10].

It is currently not understood how MAFLD and T2DM progresses from hepatic insulin resistance to whole-body insulin resistance. One school of thought is that disturbances in lipid metabolism directly influence insulin signaling, leading to impaired secretion of insulin, which contributes to the development and progression of insulin resistance and abnormal glucose metabolism that leads to T2DM [11]. While another concept is that decreased insulin secretion and insulin resistance increase lipolysis, contributing to the development of MAFLD. In addition, increased circulating insulin levels stimulate hepatic *de novo* lipogenesis in patients with MAFLD [12]. Impaired insulin signaling in adipocytes causes enhanced lipolysis that further worsens the load of lipids routing to the liver. Brown and Goldstein have suggested a selective versus total insulin resistance known as the ‘pathogenic paradox’ [13]. All of these actions might contribute to the development of MAFLD. However, more work is needed to better understand the mechanistic insights into the development of hepatic signaling in MAFLD and whether these are consistent in the latter stages of advanced liver disease, which contain less fat content [14].

The liver controls the metabolism of fatty acids through several molecular mechanisms, including the uptake and release of fatty acids, *de novo* fatty acid generation, and lipid utilization via fatty acid β-oxidation. In the first part of this review, we discuss the pathophysiology of liver disease and the molecular signaling of pathways in the development of hepatic steatosis in MAFLD. We also discuss the mechanisms that mediate the transition from MAFLD to the worsened state of non-alcoholic steatohepatitis (NASH). The second half of the review focuses on the development of therapies for MAFLD, which arise from our understanding of these pathways and other emerging therapies such as antidiabetic drugs, angiotensin II receptor antagonists, and thyroid receptor β-agonists.

## Pathophysiology of liver diseases

### Liver disease classification

**Figure 1:**
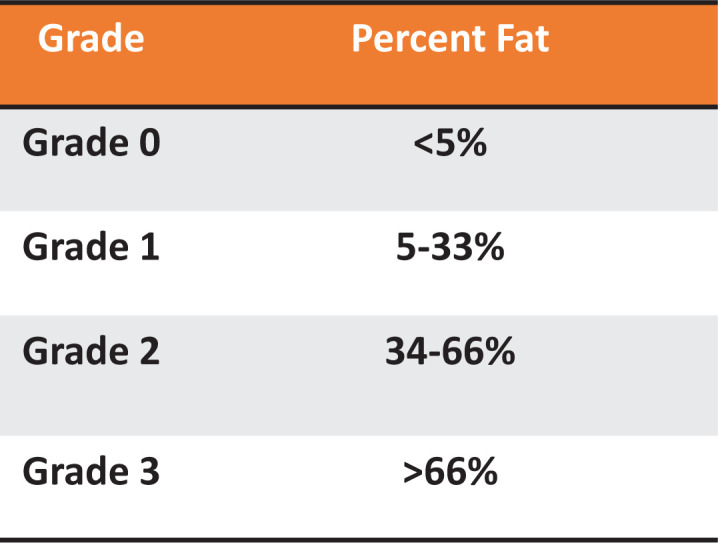
MAFLD grade of liver disease and fat content

The onset of MAFLD is characterized by fat accumulation in hepatocytes of at least 5% or more of the liver weight [15]. Hepatic steatosis is classified into various degrees based on lipid percentage in the liver cells (reviewed further in [16]). A healthy liver containing less than 5% of fat accumulation is a grade 0, mild accumulation of 5–33% fat is classified as grade 1, moderate accumulation of 34–66% of fat is grade 2, and severe accumulation with more than 66% of fat is grade 3 steatosis [17] (Figure 1). In obesity with excess calorie intake, the ability of adipose tissue to completely store fats is maximized, which leads to ectopic storage of lipids in tissues like the heart, kidneys, and liver [18,19]. The expansion of abdominal adipose tissue results in the release of cytokines and transcription factors that worsen inflammation associated with obesity [20–23]. Hepatic impairment linked to MAFLD can progress from simple steatosis, NALFD, to NASH [6]. The progression to NASH is the advanced form of MAFLD and is associated with cardiovascular-related mortality that can progress to cirrhosis, liver failure, and hepatocellular carcinoma (HCC) (see later in Figure 5).

**Figure 2:**
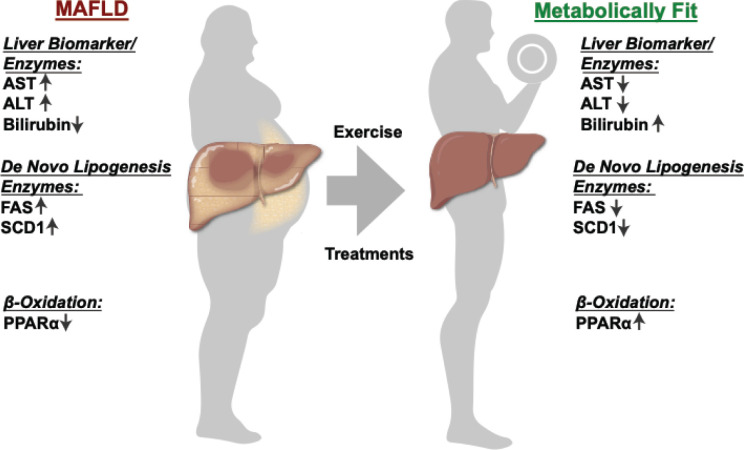
Liver enzymes levels in MAFLD and metabolically fit livers The plasma levels of aspartate aminotransferase (AST) and alanine aminotransferase (ALT) liver biomarker enzymes and bilirubin are typically used in clinics to measure the level of liver dysfunction. The level of *de novo* lipogenesis and β-oxidation are essential to regulating the liver fat content.

The liver biomarkers typically used in the clinics to measure the level of liver dysfunction are typically plasma levels of aspartate aminotransferase (AST) and alanine aminotransferase (ALT), and bilirubin [24] (Figure 2). While AST and ALT are well-established liver biomarkers for hepatocyte injury, the levels of bilirubin have been shown to not be consistent, and there is a discrepancy in the field. This is likely due to the cause of the liver dysfunction, such as fatty liver being induced by diet and excessive calorie intake or by excessive alcohol consumption [2,7,25]. Obesity and MAFLD patients typically have low plasma bilirubin levels unless they are in a stage of decompensated cirrhosis [21,26–28]. Interestingly, preclinical studies demonstrate that increasing plasma bilirubin in the obese reduces adiposity and also lowers the AST and ALT liver enzymes [29–33]. We will discuss the potential of increasing bilirubin levels in patients as a possible therapy for MAFLD later in the section on antioxidants. While AST and ALT have traditionally been utilized as markers of liver function, alterations in plasma ALT do not always accompany the histological changes associated with MAFLD [34]. The search for alternative plasma biomarkers has increased due to the fact that serum ALT levels are not always predictive of MAFLD. Markers like caspase-generated fragmented cytokeratin 18 (fCK18), β-trophin, and adipokines like adiponectin and visfatin are currently being developed as alternatives to ALT and AST for the detection of MAFLD [35–37].

### Mechanisms for the transition from MAFLD to NASH

**Figure 3:**
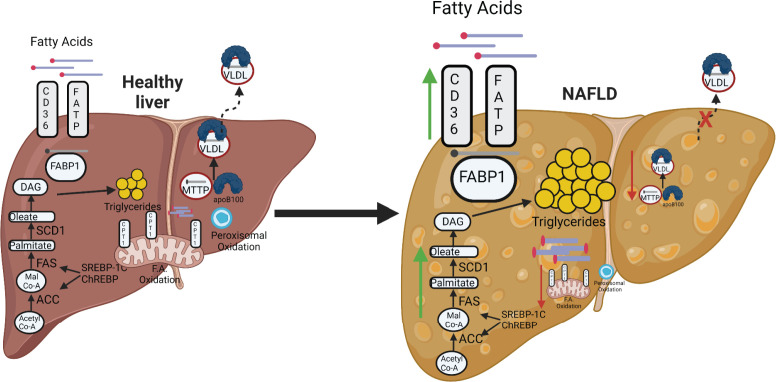
Pathways governing lipid accumulation in the liver Fatty acid uptake and *de novo* lipogenesis can be up-regulated in MAFLD (green arrows), while fatty acid export and oxidation of fatty acids by the mitochondria and peroxisomes are decreased in MAFLD (red arrows); ACC, acetyl-CoA carboxylase; DAG, diacylglycerol; FAS, fatty acid synthase; SCD1, stearoyl-CoA desaturase-1; VLDL, very-low-density lipoprotein. Created with Biorender.com

**Figure 4:**
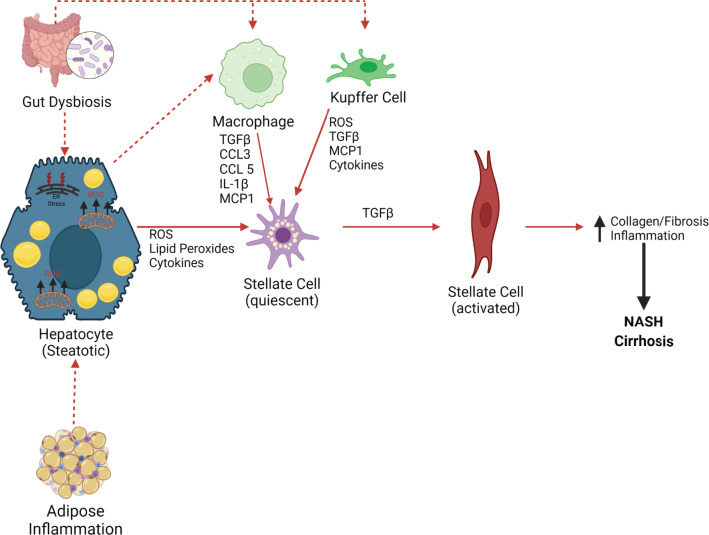
Mechanism of hepatic stellate cell activation Both adipose tissue inflammation and gut dysbiosis can contribute to hepatocyte steatosis. Hepatocyte steatosis increases endoplasmic reticulum (ER) stress and mitochondrial ROS production, which alters hepatic cytokines resulting in stellate cell activation. Hepatic steatosis and gut dysbiosis also lead to the activation of hepatic macrophages and Kupffer cells that further activates stellate cells to increase collagen synthesis resulting in fibrosis and inflammation and promoting NASH; CCL3/5, chemokine (C-C motif) ligands 3/5; ER, endoplasmic reticulum; IL-1β, interleukin 1β; MCP1, monocyte chemoattractant protein-1; NASH, non-alcoholic steatohepatitis; ROS, reactive oxygen species; TGFβ, transforming growth factor-β. Created with Biorender.com

There are several potential mechanisms responsible for the transition from MAFLD to NASH [38,39]. A combination of both extrinsic and intrinsic factors mediates this transition. Adipose tissue inflammation can contribute to insulin resistance and excess release of free fatty acids by lipolysis, which causes hepatic lipid accumulation (Figure 3). The gut microbiota might also play a role in gastrointestinal physiology and the regulation of the intestinal immune system [28]. Changes in the quality and quantity of the gut microbiota, which are associated with deleterious health effects, are referred to as dysbiosis. Studies in mice demonstrate that transferring gut bacteria from humans with MAFLD to lean mice increases insulin resistance and inflammation in response to a high-fat diet, and this results in a greater increase in hepatic steatosis [28,40]. Alterations in the gut microbiota can also result in changes in intestinal permeability to agents like bacterial lipopolysaccharide, which activates Kupffer cells to secrete proinflammatory cytokines, which activates fibrosis pathways by hepatic stellate cells (HSCs) (Figure 4) [41]. Steatosis in hepatocytes can potentially result in the activation of HSCs via several mechanisms [39]. First, lipid accumulation in hepatocytes induces endoplasmic reticulum (ER) stress and increases reactive oxygen species (ROS) production that activates fibrotic pathways, such as the inclusion of fructose with a high-fat diet [39]. Both of these conditions can result in the release of ROS and lipid peroxidation products as well as cytokines, all of which send signals for injury to the liver, such as tumor growth factor-β (TGFβ) that induces HSC activation [39] (Figure 4). Hepatic macrophages produce cytokines and chemokines that directly influence HSC activation, including TGFβ, IL-1β, MCP1, CCL3, and CCL5 [42]. Proinflammatory cytokines such as TGFβ and IL-1β activate HSCs [39]. CCL3 and CCL5 promote hepatic fibrosis through binding with CCR1 and CCR5 on HSCs. Activation of HSCs results in the expression of α-smooth muscle actin (αSMA) [39] and S100 calcium-binding protein A6 (S100A6), the formation of stress fibers, and the deposition of extracellular matrix components. The secretion of profibrotic cytokines promotes the generation of fibrosis, and this interaction with fibrotic tissue further activates HSCs and promotes NASH (Figure 4). Recent data suggest that growth factors such as stem cell growth factor β (SCGF-β), granulocyte-macrophage colony-stimulating factor (GM-CSF), and macrophage colony-stimulating factor (M-CSF) can worsen insulin resistance in males with MAFLD [43].

While MAFLD is widely viewed as a hepatic insulin-resistant disease, most emerging possible therapeutics are to improve hepatic insulin resistance. However, it should be noted that a recent study showed that as the liver disease worsens to NASH and cirrhosis, there could be a change in the insulin signaling in the liver from resistance to sensitivity [39]. An intriguing study by Yen et al. found that patients with liver cirrhosis and insulin-resistant T2DM that use insulin injection therapeutics had significantly elevated risks of death, as well as hypoglycemia, and liver- and cardiovascular complications, compared with those that have the same conditions but did not use insulin injections [44]. Others have also published works indicating that the advanced stages of liver disease might be insulin sensitive for some tissues, such as cirrhotic patients were observed to have greater insulin binding in erythrocytes due to increased insulin receptors on the surface [45]. This work was corroborated by others who showed higher insulin responsiveness in erythrocytes of cirrhotic patients [46–48]. However, not all tissues respond the same. Some studies show reduced insulin receptor activity in immune cells [49] and adipocytes [50]. It is possible that the insulin sensitivity in the liver changes with the progression of the disease and also that other tissues may not be affected in the same manner. This is likely due to hepatocyte death that occurs in the later stages of hepatic disease and HSC activation, which indicates a reorganization of the liver that comprises different cell types in the end stages. Therefore, therapeutics targeting insulin resistance in MAFLD may not be effective in advanced liver diseases with worsened states. Future studies might take a closer analysis of hepatic insulin signaling in the advanced stages of liver disease to determine whether these changes might be occurring, especially since this could impact the life span of the patients. Herein, we will discuss the molecular signaling and mechanistic details that lead to events in MAFLD and the advantages and disadvantages of some of the possible therapeutics.

## Molecular mechanisms of lipid accumulation in mafld

### Excessive lipid uptake

#### FABP1

Fatty acid-binding protein 1 (FABP1), originally referred to as liver-FABP or L-FABP, is almost exclusively expressed in the liver. FABP1 transports fatty acids between organelles and can also bind cytotoxic free fatty acids and facilitate their oxidation or incorporation into triglycerides [51]. Genetic deletion of FABP1 significantly attenuates fasting-induced increases in hepatic triglyceride uptake and oxidation [52]. FABP1 knockout mice are protected against dietary-induced hepatic steatosis; however, altered energy utilization exhibited by the global loss of FABP1 is a confounding factor in these experiments [53]. More specific hepatocyte targeting using adenoviral vectors to knock down FABP1 demonstrated protection from dietary-induced hepatic steatosis and inflammation [54]. A recent study in human MAFLD patients found higher levels of plasma FABP1 correlated with the degree of hepatic steatosis [55]. However, whether serum FABP1 levels correlate to expression in the liver remains controversial as FABP1 is also expressed in the proximal tubule of the kidney, which may contribute to the plasma levels [56]. Currently, there are no therapeutics specifically targeting FABP1 that are used for MAFLD. More studies on the impact of antagonizing FABP1 in humans and how they affect metabolic dysfunction are needed.

#### FATP and CD36

Lipid levels in the liver are regulated by the intricate interaction between hepatic lipid uptake and *de novo* lipogenesis versus lipid β-oxidation and fatty acid export (Figure 3). Hepatic lipid uptake is controlled via the actions of fatty acid transport proteins (FATPs) and the cluster of differentiation 36 (CD36). FATP isoforms 2 and 5 are the major isoforms present in the liver [51]. Genetic deletion of FATP5 resulted in lower hepatic triglyceride and free fatty acid content in mice on a normal fat diet [57]. Adenoviral knockdown of FATP5 in mice with established high-fat diet-induced MAFLD restored insulin sensitivity; however, this improvement in hepatic function was associated with decreased caloric intake and a reduction in body weight of mice administered an adenovirus containing a short hairpin ribonucleic acid (shRNA) targeting FATP5 [58]. In humans, a polymorphism in the FATP5 promoter results in increased FATP5 expression, which correlates with higher hepatic steatosis in male MAFLD patients [59]. The levels of CD36 protein are increased in the liver in response to high-fat diet feeding [60,61]. Human MAFLD patients have increased hepatic CD36 expression, which was associated with high levels of hepatic fat content [62]. Adenoviral overexpression of CD36 increased hepatic lipid uptake and steatosis in mice fed a normal fat diet [60]. A relationship between hepatic lipotoxicity, FATP5, and CD36 in the liver exists; however, the specific mechanisms by which these proteins are altered in MAFLD, as well as their impact on the progression to NASH, is undefined at the present time [63]. Future studies to elucidate if targeting these mechanisms is a useful therapeutic are needed.

### *De novo* lipogenesis

Three essential enzymes, acetyl-CoA carboxylase (ACC), fatty acid synthase (FAS), and stearoyl-CoA desaturase-1 (SCD1), regulate *de novo* lipogenesis in the liver (Figure 3). ACC governs the conversion of acetyl-CoA to malonyl-CoA, which is converted to palmitate by FAS [64]. SCD1 catalyzes the formation of oleate and palmitoleate from stearoyl-CoA and palmitoyl-CoA [64]. These newly generated fatty acids can undergo a wide variety of biological modifications, including desaturation, elongation, and esterification, before either being stored as triglycerides or exported as VLDL particles [42,65]. The levels of these two proteins can increase hepatic steatosis and hypertriglyceridemia [64]. Moreover, increased production of palmitate may lead to steatohepatitis through increases in inflammation and apoptosis [66]. Two transcription factors that regulate FAS and SCD1 to control *de novo* lipogenesis are the sterol regulatory element-binding protein 1c (SREBP1c) and carbohydrate regulatory element-binding protein (ChREBP). Hepatocyte-specific overexpression of SREBP-1c results in the upregulation of key enzymes in *de* novo lipogenesis resulting in hepatic lipid accumulation [67]. ChREBP is required for normal lipogenic response following the ingestion of carbohydrates such as sucrose and fructose [68]. ChREBP knockout mice exhibit decreased levels of lipogenic genes such as ACC and FAS in response to a high starch diet suggesting that ChREBP is the transcriptional mediator of carbohydrate induction of lipogenesis in the liver [69]. Liver-specific inhibition of ChREBP in ob/ob mice via adenovirus shRNA suppression improves hepatic steatosis by decreasing lipogenesis resulting in lower levels of plasma triglycerides and nonesterified fatty acids [70]. Enhanced *de novo* lipogenesis is responsible for a portion of the lipid accumulation in MAFLD and the buildup of toxic lipid species such as ceramides that may promote the transition to NASH. However, care must be taken when proposing blocking specific enzymes in the *de novo* lipogenesis pathway, as this could promote the accumulation of more toxic lipids [64]. Further studies are needed before this pathway should be considered as a potential therapeutic target for the treatment of MAFLD as there may be major side effects from blockade of normal lipid processing.

### β-Oxidation of fatty acids

Oxidation of fatty acids primarily occurs in the mitochondria; however, oxidation of very long-chain fatty acids commences in the peroxisomes and then is finally processed in the mitochondria (Figure 3) [64]. In cases of lipid overload, as seen in obesity, ω-oxidation by cytochrome P450 enzymes also contributes to fatty acid oxidation. However, this pathway’s oxidation of fatty acids generates large amounts of ROS, which promote inflammation and NASH progression [71]. The entry of fatty acids into mitochondria depends on carnitine palmitoyltransferase 1 (CPT1), situated in the outer mitochondrial membrane. One of the major regulators of CPT1 is the peroxisome proliferator-activated receptor-α (PPARα) [31,32,72,73]. Activation of PPARα induces transcription of genes related to fatty acid oxidation in the mitochondria (CPT1), peroxisomes (acyl-CoA oxidase, ACOX), and cytochromes (Cyp4A family) [31,74,75]. Hepatocyte-specific PPARα knockout mice exhibit marked steatosis, inflammation, and suppressed levels of CPT1 as compared with wild-type mice in response to dietary-induced obesity [42]. Glycogen synthase kinase 3β (GSK3β) regulates levels of PPARα in the liver through phosphorylation at serine-73 (Ser73) [73]. Ser73 phosphorylation enhances the ubiquitination of PPARα, resulting in increased protein turnover and decreased activity [73]. In humans, the expression of PPARα decreases with increasing MAFLD activity score and fibrosis score [74]. Targeting this pathway alone or in combination with the PPARγ isoform for the potential treatment of MAFLD will be discussed further below.

### Export of hepatic lipids

The export of triglycerides is another important pathway in the regulation of hepatic lipid content. The key components of this process are apolipoprotein B100 (apoB100) and microsomal triglyceride transfer protein (MTTP) (Figure 3). Very low-density lipoprotein (VLDL) particles are formed in the endoplasmic reticulum (ER), where MTTP catalyzes the lipidation of apoB100 [29,31]. The VLDL particles are then transferred to the Golgi apparatus, where they mature and are subsequently secreted into the bloodstream via ApoB100-mediated mechanisms. Moderate exposure to fatty acids increases apoB100 secretion; however, increased levels of fatty acids promote ER stress which subsequently inhibits apoB100 secretion, promoting steatosis [76]. Genetic defects in MTTP affect hepatic triglyceride export and cause MAFLD. In a study of MAFLD susceptibility in the Han Chinese population, genetic polymorphisms in the MTTP gene both increased and decreased the risk of MAFLD [77]. Overexpression of MTTP normalized VLDL secretion and reduced hepatic triglyceride levels in a mouse genetic model of MAFLD [78]. Diminished levels of apoB100 result in decreased secretion of VLDL. NASH patients have lower apoB100 synthesis rates indicating that lower lipid transport could contribute to advanced steatosis in these patients [79]. Taken as a whole, alterations in MTTP and apoB100, especially in conditions of lipid overload, may result in steatosis and lipotoxicity that promotes the progression of MAFLD.

## A mechanistic view of therapies for MAFLD

### First-line therapies

**Figure 5:**
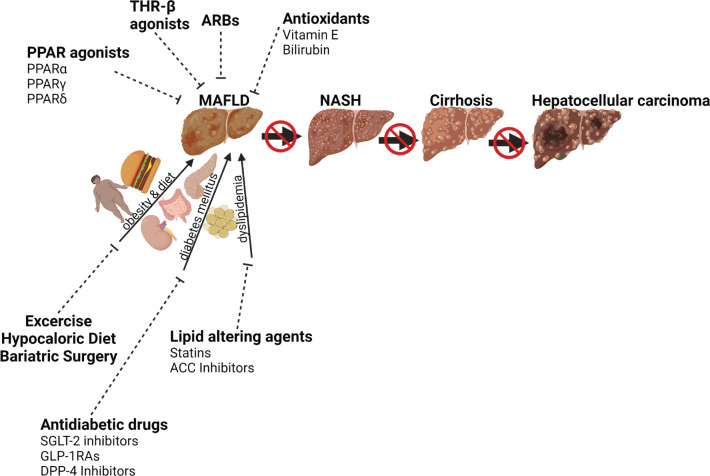
Influence of current and emerging therapies on MAFLD Risk factors such as obesity, diet, diabetes mellitus, dyslipidemia, oxidative stress, inflammation, and apoptosis stimulate MAFLD, which can progress to liver fibrosis, cirrhosis, and hepatocellular carcinoma. Lifestyle intervention (diet and exercise) and therapeutic interventions inhibit fatty liver diseases; ARB, angiotensin receptor blocker; DPP-4, dipeptidyl peptidase-4; FGF, fibroblast growth factor; GLP-1RAs, glucagon-like peptide-1 receptor agonists; PPAR, peroxisome proliferator-activated receptor; SGLT-2, sodium-glucose cotransporter-2; THR-β, thyroid hormone receptor-β. Created with Biorender.com

The most commonly recommended treatment for MAFLD is exercise and dietary restriction (Figure 2) [80]. While there are no FDA-approved therapies specifically for MAFLD, we describe in Figure 5 and below some of the current therapies being used and developments in research that may lead to the first approved treatment regimens.

### Antidiabetic drugs

In treating MAFLD, medications targeting diabetes, a disease often associated with MAFLD development, are longstanding (Figure 5). Metformin, a biguanide, is the first-line oral antidiabetic therapy prescribed to T2DM patients functions to lower blood glucose, and this may be beneficial to MAFLD patients with T2DM [5]. Bugianesi et al. determined that metformin had a larger effect on improving aminotransferase levels in patients with MAFLD than in another treatment group with vitamin E [5,81]. The mechanism of action of metformin in the liver involves the activation of AMP-activated protein kinase (AMPK), a mechanism that may be responsible for its wide range of metabolic benefits [82]. Studies have shown that AMPK inhibits ACC, thereby reducing malonyl CoA synthesis, which in turn increases fatty acid oxidation in the mitochondria [73,83]. In MAFLD induced by a high-fat diet, metformin directly decreased fat deposition and inhibited inflammation in the liver by increasing phosphorylation of liver AMPK and ACC but decreased lipogenic enzymes and proinflammatory cytokines [84]. Furthermore, the reversal effect of metformin on insulin resistance and hepatic steatosis is autophagy-dependent via the transcriptional factor EB, a potent regulator of autophagy [85]. Although metformin has been documented in some studies to be beneficial in MAFLD and chronic hepatitis C patients [86,87], its use has been inadvisable in patients with cirrhosis due to increased risk of lactic acidosis [88,89]. Also, its long-term use has been linked with vitamin B12 deficiency [15,90]. Metformin use can also be associated with abdominal discomfort, anxiety, and interaction with other classes of drugs. While metformin reduces serum aminotransferases and insulin resistance in several preclinical studies, several meta-analyses determine no significant improvement in liver histology in MAFLD and NASH patients when treated with metformin therapy [91–93].

Sodium-glucose cotransporter-2 (SGLT2) inhibitors are another class of drugs approved for the treatment of T2DM. SGLT2 inhibitors decrease renal reabsorption of glucose, helping to improve glycemic control. Several studies have evaluated the impact of SGLT2 inhibitors on MAFLD and have found improvements in AST and ALT liver enzymes, as well as in hepatic fat accumulation [5]. SGLT2 inhibitors have been reported to reduce body fluid and body weight; hence, they can be recommended in obese patients with MAFLD [94]. Shiba et al. reported that canagliflozin treatment for 20 weeks delayed the onset of NASH and its progression to hepatocellular carcinoma in a mouse model of human NASH [95]. Likewise, in a human study, treatment with canagliflozin resulted in reduced liver enzymes [ALT, AST, alkaline phosphatase (ALP), and gamma-glutamyl transferase (GGT)], body weight, and an increase in bilirubin [96]. Empagliflozin, another SGLT2-inhibitor, reduces hepatic fat content and significantly improves steatosis and liver fibrosis, as well as reduces AST and ALT in MAFLD patients with or without the condition of T2DM [97–99]. Empagliflozin decreased the expression of hepatic inflammatory genes such as TNF-α, interleukin-6, and monocyte chemoattractant protein-1 (MCP-1) in a mice model of NASH, and when used in combination with linagliptin, a DPP-4 inhibitor, both treatments reduced expression of mRNAs for genes related to fatty acid synthesis, reduced collagen deposition and decreased the expression of αSMA, which is an indicator of fibrosis [39,100]. Empagliflozin also reduced insulin resistance and attenuated diet-induced activation of NLRP-3 inflammasome and triglyceride in the liver [101]. Ipragliflozin reduced plasma and liver biomarkers of oxidative stress such as thiobarbituric acid reactive substances and protein carbonyl and inflammatory markers in T2DM mice with NASH [102]. SGLT2 inhibitors have several side-effects that may limit their use including risk of dehydration, potential for the development of urinary tract infections, and development of ketoacidosis. SGLT2 inhibitors are promising new therapies for MAFLD and other cardiovascular diseases such as congestive heart failure; however, it is unclear if they have a direct effect on hepatic steatosis or work through effects on body weight and insulin sensitivity.

Another class of drugs benefiting MAFLD patients is glucagon-like peptide-1 (GLP-1) receptor agonists (GLP-1RAs). Examples of these drugs are exenatide, liraglutide, dulaglutide, and semaglutide. These drugs are commonly used to treat diabetes, and they mimic the action of an incretin hormone called GLP-1, which is released from the L cells of the small intestine after meals to stimulate insulin secretion, reduce glucagon production, and retard gastric emptying [103]. Hence, this antidiabetic drug class induces glucose-mediated insulin secretion and reduces glucagon secretion, promoting weight loss [5]. Interestingly, there is down-regulation of GLP-1 receptors in MAFLD [104]. In preclinical studies, GLP-1 analogs have been demonstrated to inhibit MAFLD by inhibiting NLRP3 inflammasome via enhancement of autophagy/mitophagy pathways, inhibiting macrophage recruitment and activation, and increasing antioxidant defense in the liver [105]. These GLP-1RAs improve insulin sensitivity by reducing JNK phosphorylation and increasing the expression and activity of PPARγ [106]. Several randomized controlled trials (RCTs) have documented its efficacy in reducing liver enzymes, body weight, and hepatic content. In a multicenter clinical trial of 76 MAFLD patients, 24-week treatment with exenatide reduced hepatic steatosis; however, these changes were associated with reductions in body weight, visceral fat, and fasting glucose levels [107]. Taken together, GLP-1RAs have the potential to be developed into therapies for MAFLD as they act on multiple pathways (hepatic lipogenesis and β-oxidation, ER stress, and oxidative stress) and also have beneficial effects to lower body weight and improve insulin sensitivity. GLP-1RA use is associated with minor side-effects such as nausea. All these taken together, GLP-1RAs delay the progression of MAFLD by inhibiting inflammation, oxidative stress, insulin resistance, enzymes involved in hepatic lipogenesis, and enhancing the autophagy/mitophagy pathway, as well as enzymes involved in β-oxidation and offer the potential to be useful therapies.

DPP-4 inhibitors, also called gliptins, are potential medications for the treatment of MAFLD (Figure 5) that work by obstructing the activities of dipeptidyl peptidase-4 (DPP-4) to elevate incretin levels and reduce glucagon release. This action consequently decreases gastric emptying, lowering hepatic glucose production, increasing insulin exocytosis, and fatty acid oxidation in the liver [108]. Sitagliptin, a gliptin drug, was reported to attenuate the expression of SREBP-1c, SCD-1, and FAS which are involved in *de novo* lipogenesis, and it up-regulated the expression of PPARα in the liver [109]. The SIRT1/AMPK pathway also mediates the anti-steatotic actions of sitagliptin in the liver [110]. Although several studies show that sitagliptin reduces hepatic lipid accumulation in animal models, there are conflicting results in human MAFLD patients. Sitagliptin therapy was initially reported to suppress liver enzymes, body weight, and hepatocyte ballooning in diabetic patients with NASH [111]. However, a follow-up study by Joy et al. showed no significant improvement in the liver fibrosis score, MAFLD activity score, lipid profile, or liver enzymes in patients with biopsy-proven NASH that received sitagliptin for 24 weeks [112]. There are othergliptin drugs that act by improving insulin resistance and hepatic inflammation that could be beneficial in MAFLD patients [113]. Randomized clinical trials of this class of DPP-4 inhibitors are needed in MAFLD patients. In addition, use of DPP-4 inhibitors is associated with upper respiratory tract infection, headaches, and a potential for hypoglycemia especially if used in concert with insulin.

Thiazolidinediones, PPAR- γ agonists, are insulin sensitizers used in the management of diabetes and for the treatment of MAFLD. The two major thiazolidinediones that have been used to treat diabetes are pioglitazone and rosiglitazone. In human studies, pioglitazone improved hepatic steatosis, ballooning necrosis, and inflammation; however, no changes in fibrosis were reported [114,115]. Small clinical trials of rosiglitazone demonstrated the overall tolerance as well as reductions of serum markers ALT and AST and lowering of plasma glucose, insulin, and HbA1c [116,117]. While rosiglitazone treatment was generally well-tolerated in these small clinical trials, effects such as weight gain, fluid retention, osteopenia, prostate and pancreatic cancer, and cardiovascular events have been ascribed to thiazolidinedione use [118].

### Inhibiting *de novo* lipogenesis

ACC is an important enzyme in *de novo* lipogenesis and an emerging target for the treatment of MAFLD (Figure 5). ACC is present in two isoforms in the liver: ACC1 and ACC2. The use of dual inhibitors of ACC1 and ACC2 was effective in preventing hepatic steatosis in mouse models of dietary-induced MAFLD. However, Goedeke et al. reported that dual inhibition of ACC1 and ACC2 also resulted in plasma hypertriglyceridemia from a combination of increased hepatic very-low-density lipoprotein production and a decrease in triglyceride clearance by lipoprotein lipase (LPL) [119]. Selective ACC1 inhibitors have also been utilized to treat MAFLD in a mouse model of genetic fatty liver [120]. Clinical trials utilizing dual ACC inhibitors alone or in combination with diacylglycerol O-acetyltransferase 2 (DGAT2) are currently underway in adults with MAFLD [121]. In these studies, ACC inhibition resulted in decreases in hepatic steatosis as measured by magnetic resonance imaging (MRI); however, like studies in animals, ACC inhibition resulted in a significant increase in plasma triglyceride levels [121]. Treatment with both ACC and DGAT2 inhibitors also lowered hepatic steatosis but did not result in any significant changes in plasma triglyceride levels [121]. These results suggest that the use of ACC inhibitors, when coupled with drugs that inhibit triglyceride synthesis, may be effective therapies to reduce hepatic steatosis in MAFLD patients.

### Targeting excess lipid uptake

The excess lipid uptake mediated through FATP and CD36 contributes to hepatic steatosis in NAFLD (Figure 3). The FATP2 inhibitor, Grassofermata/CB5, was shown to inhibit palmitate-mediated lipid accumulation in HepG2 cells; however, its efficacy has not been tested in animal models [122]. While drugs specifically targeting these pathways are still in development, there are several clinically utilized drugs affecting this pathway that could be beneficial in NAFLD. For example, the PDE4 inhibitor, roflumilast, was clinically approved for the treatment of chronic obstructive pulmonary disease and may be beneficial in treating MAFLD. Roflumilast treatment of dietary-induced obese mice reversed hepatic lipid accumulation [123]. The antitumor and immunosuppressive drug rapamycin [124] was shown to reduce lipid accumulation in 3T3-L1 cells [125] and improve hepatic steatosis by reducing CD36 expression in mice fed a high-fat diet [126]. Rapamycin acts through inhibition of the mammalian target of rapamycin (mTOR) pathway. It also has been shown to suppress hepatic CD36 translational efficiency, decreasing CD36 levels and alleviating hepatic steatosis [126]. However, rapamycin also binds to the FKBP12 [125,127] and has immunosuppressive effects that have been linked to glucose intolerance in mice [128]. There are more studies needed for a better understanding of how targeting these mechanisms might be beneficial, and the efficacy of the compounds should be considered.

### Targeting hepatic β-oxidation

#### PPARα agonists

PPARα is highly expressed in the liver [23,129], where it is vital in triglyceride hydrolysis, fatty acid catabolism, glycerol metabolism, and ketogenesis [130,131]. Studies in mice show that the loss of PPARα in the liver results in simple steatosis on a normal chow diet [129], which is worsened on a high-fat diet [42,132]. Likewise, systemic loss of PPARα coupled with a trans fatty acid-rich diet leads to hepatic steatosis [133]. Fibrates, which target PPARα, are prescribed clinically to lower serum lipids and also have beneficial effects on hepatic steatosis in animal models. In addition, existing data show that fibrate treatment prevents stroke, myocardial infarction, and cardiovascular death [134–136]. The PPARα agonist fenofibrate increases both uptake and β-oxidation of FAs which is for lipid utilization [64]. Fenofibrate protects the liver against MAFLD (Figure 5) by improving insulin sensitivity and by exerting antioxidant, anti-inflammatory, and anti-atherosclerotic effects [137–143]. In mice lacking phosphatidylethanolamine *N*-methyltransferase (PEMT), fenofibrate treatment completely prevented MAFLD development and partially reversed steatosis and fibrosis in already established MAFLD in this model [144]. Similarly, administration of fenofibrate for 5 days decreased hepatic lipid and glycogen storage in neonatal glucose-6-phosphatase α knockout mice via promotion of β-oxidation of fatty acids and stimulation of hepatic expression of acyl-CoA dehydrogenases [145]. Pemafibrate (K-877), another selective PPARα agonist, improved methionine–choline-deficient diet-induced MAFLD by increasing the expression of hepatic fatty acid oxidation genes in mice [146]. In a phase 3 clinical study, pemafibrate treatment significantly reduced plasma triglycerides in at-risk patients and had a superior safety profile as compared with fenofibrate [147]. It should be noted that fibrates have minimal clinical use as they have varied effects and, at times, show very little response in some patients. Fibrate use is also associated with side effects such as abdominal pain, constipation, headaches and muscle cramps. Preclinical animal studies show that this might be related to estrogen suppression of PPARα, reducing the effectiveness of fibrates in females [148–151]. Hinds et al. showed that female mice have significantly less PPARα expression in the adipose compared with males [23]. Only the males in this study were shown to be affected by the adipose-specific loss of PPARα [23]. This suggests that more studies are needed to better understand the varied effects by which estrogen status affects PPARα expression.

#### Dual/Pan PPAR-alpha-gamma agonists

The development of MAFLD involves numerous processes, and some novel PPAR-agonists having effects on two or more PPAR isoforms have been shown to be effective in the treatment of MAFLD (Figure 5). Such drugs are classified as dual/pan-PPAR agonists. Saroglitazar is a dual agonist of PPARα and PPAR-γ that is in multiple phase 3 clinical trials. Saroglitazar use is not associated with the side effects (weight gain, edema) linked with activation of PPARγ agonists like pioglitazone [152,153]. In a mouse model of dietary-induced MAFLD, saroglitazar treatment lowered body weight, insulin resistance, triglycerides, total cholesterol, ALT, and improved hepatic steatosis and fibrosis [154]. Compared with pioglitazone and fenofibrate, saroglitazar improved steatosis, fibrosis, and NASH to a greater extent by influencing serum inflammatory cytokines and adiponectin levels in rats fed a high-fat diet [155]. In human clinical trials, saroglitazar reduced ALT levels and improved fatty liver in MAFLD patients with diabetic dyslipidemia [156]. Goyal et al. documented that hepatic enzymes and liver stiffness were improved by saroglitazar in patients with diabetic dyslipidemia and MAFLD [9]. The results from these clinical trials demonstrate that saroglitazar is a promising therapeutic option for MAFLD patients. Elafibranor (GFT-505) is another dual agonist of PPARα and PPARδ [130]. Several studies have demonstrated the role of elafibranor in different diet-induced obese mouse models of NASH. Elafibranor reduced body weight, liver enzymes, histopathological scores of hepatic steatosis and inflammation, and attenuated fibrosis severity in NASH models of C57BL/6J mice and leptin-lacking *Lep*^ob/ob^ fed on high fat, fructose, and cholesterol diet [157]. In another study, elafibranor reduced hepatic steatosis and inflammation and prohibited the progression of fibrosis in a mouse genetic model that exhibited histopathological characteristics of NASH with similar molecular pathways observed in NASH patients [158]. In a human study, abdominally obese insulin-resistant male patients treated with elafibranor for 2 weeks showed reduced serum lipids, improved liver enzymes, and enhanced hepatic insulin sensitivity [159]. In a study carried out for 52 weeks in Europe and United States, patients with NASH without cirrhosis treated with two doses of elafibranor showed resolution of NASH without worsening of fibrosis. In addition, the higher dose of elafibranor reduced liver enzymes, lipids, glucose profiles, and markers of systemic inflammation compared with placebo-treated patients [160]. However, a recent phase 3 clinical trial (NCT02704403, RESOLVE-IT) evaluating the use of elafibranor versus placebo in the treatment of NASH was terminated due to lack of efficacy.

Lanifibranor (IVA337) is a pan-PPAR agonist that activates the three PPAR isoforms (α, γ, δ) proportionately [130]. Lanifibranor controlled body weight gain, improved insulin sensitivity, attenuated hepatic steatosis, inflammation, ballooning, and fibrosis, as well as inhibited the expression of pro-fibrotic and inflammatory genes, and increased the expression of β-oxidation genes in studies with various preclinical models of NASH [161]. Lanifibranor improves insulin sensitivity, lipid profile, inflammation, and fibrosis, and it has been reported to have a good safety profile [162]. In a recently completed phase 2 clinical trial, lanifibrnor decreased the activity part of the Steatosis, Activity, Fibrosis (SAF) score, SAF-A in patients with noncirrhotic NASH [163].

### Angiotensin receptor blockers (ARBS)

Angiotensin receptor blockers (ARBs), also known as angiotensin II receptor antagonists, are traditionally used to treat hypertension, heart failure, and chronic kidney disease but have potential in the treatment of MAFLD (Figure 5). In animal models, ARB treatment lessened hepatic steatosis and fibrosis, reduced markers of inflammation, and improved hepatic mitochondrial function [164,165]. ARB treatment attenuated transhepatic pressure gradients and intrahepatic vascular resistance in the high-fat, high-fructose diet-induced NASH model [166]. ARB treatment was also found to reverse mitochondrial dysfunction and reduce the proinflammatory M1 macrophage population [165]. Studies have shown that increasing the M1 macrophage population in the liver of mice induces fat accumulation [167]. However, a confounding study reported that dietary intervention but not losartan reversed obesity, insulin resistance, autophagic flux, and histological features of NASH [168]. ARBs also elevate circulating adiponectin levels which can exert an antifibrotic effect, improving both MAFLD and NASH [169,170].

Several phase 2 clinical trials have examined the efficacy of ARBs in MAFLD. In a phase 2 clinical trial study, MAFLD pediatric patients treated with losartan for 8 weeks showed improved ALT, AST, and insulin resistance compared with placebo [171]. In the fatty liver protection trial by telmisartan or losartan study (FANTASY), telmisartan showed more beneficial effects in improving fatty liver than losartan in hypertensive MAFLD patients with T2DM [172]. This is likely due to the fact that telmisartan activates PPARγ [173]. ARBs not only are effective anti-hypertensive drugs, but they may also prove beneficial in the treatment of patients with MAFLD. More studies are needed to better understand the potential uses of the ARBs in treating MAFLD.

### Thyroid hormone receptor-β agonist

Resmetirom (MGL-3196) is an orally administered selective thyroid hormone receptor-β (THR-β) agonist. The thyroid hormone acts via THR-β in the liver to increase lipid metabolism and prevent pathological lipid accumulation in the liver. Several studies have shown the involvement of THR-β in MAFLD/NASH metabolic pathways [174–176]. In a mouse model of NASH and fibrosis, resmetirom improved MAFLD activity score and decreased hepatic fibrosis by lowering the content of αSMA and down-regulating genes involved in fibrogenesis such as collagen 1α1, lysyl oxidase-like 2, and hydroxysteroid 17-β dehydrogenase 13 [176]. THR-β agonists improve mitochondrial β-oxidation and reduce hepatic lipid accumulation in NASH patients [177]. In a phase 2 trial, resmetirom reduced hepatic fat accumulation after 12 weeks and 36 weeks of treatment in patients with NASH [178]. Presently, there are two ongoing phase 3 clinical trials; one is to evaluate the efficacy and safety of MGL-3196 in NASH patients with advanced fibrosis (MAESTRO-NASH), and the other is to evaluate the safety of MGL-3196 in MAFLD patients (MAESTRO-MAFLD-1) [175]. These clinical studies should reveal the usefulness of these compounds and if targeting THR-β is useful for treating MALFD. Resmetirom use is associated with mild side effects such as transient nausea.

### Antioxidants

Antioxidants such as Vitamin E has been used for MAFLD treatment (Figure 5) in hopes of combating the ROS and oxidative stress that is known to occur [179]. A meta-analysis by Abdel-Maboud et al. analyzing 1317 patients from 15 randomized controlled trials showed that vitamin E was efficacious at reducing MAFLD activity score and AST and ALT liver enzymes [180]. There was a clinical trial (NCT01792115) to determine the dosage of vitamin E combined with exercise that provides the best protection. The study only found modestly reduced liver fat content with the highest dose. Another antioxidant, bilirubin [181], has gained attention for its recently discovered role that it is also a hormone that activates PPARα [21,38,182,183]. Bilirubin strongly reduces oxidative stress in hepatocytes [184] and in other cell types [185–187]. Preclinical studies show that bilirubin suppresses hepatic fat content in obese mice and lowers hepatocyte injury markers AST and ALT [29,31,32]. Plasma bilirubin levels are lower in patients with MAFLD [188–190]. However, other studies have not found that bilirubin levels are changed in MAFLD [191]. It likely depends on the metabolic state of the patients, as patients exhibiting mildly elevated (>12 μM) bilirubin levels were shown to have significantly fewer metabolic disorders such as NAFLD, obesity, or T2DM [192–200]. This is supported by a study by Lin et al. that found that obese children with the UGT1A1*6 variant with slightly elevated plasma bilirubin levels were less likely to be diagnosed with MAFLD [201]. One of the current therapeutics used for MAFLD is dietary restriction and exercise. Interestingly, plasma bilirubin levels are increased during exercise [181,202], which may be one of the mechanistic actions that exercise improves liver fat content. However, more studies are needed to better understand this aspect. Bilirubin can also impact hepatic steatosis by acting as an activator of the nuclear hormone receptor PPARα [32,203]. Bilirubin is able to increase the levels of CPT1 and Cya4A family isoforms to increase the oxidation of fatty acids [32]. Treatment with bilirubin nanoparticles also attenuates dietary obesity-induced hepatic steatosis and increases plasma ketone levels [29]. Other options of possibly increasing bilirubin levels in the blood are to target the enzyme that conjugates it, UGT1A1 [31,204]. More work is needed to reveal if antioxidants might be useful for treating MAFLD. It could be that they might be better in combination therapy in which one reduces ROS and the other burns fat, and they may be more efficacious together at reducing MAFLD.

## Conclusions

MAFLD is becoming a common cause of chronic liver disease and significantly impacts our healthcare system. With very few FDA-approved drugs explicitly for the treatment of MAFLD, patients must rely on weight loss from lifestyle changes or medications to treat comorbidities such as diabetes and hypertension. It is clear from the scope of this review and others that advances in our understanding of the molecular pathways regulating hepatic lipid accumulation can lead to promising therapies for MAFLD [205]. In addition, several drugs used for conditions like diabetes, obesity, and hypertension are being repurposed for use in the treatment of MAFLD (Figure 5). By systematic dissection of the pathways responsible for hepatic lipid accumulation and the progression of MAFLD, new therapeutic targets for MAFLD should be identified. It is hopeful that the emerging therapies will be successful in attenuating the emerging epidemic of MAFLD and its associated pathologies.

## Data Availability

All data are included within the main manuscript file.

## Open Access

Open access for this article was enabled by the participation of University of Mississippi in an all-inclusive *Read & Publish* agreement with Portland Press and the Biochemical Society.

## Abbreviations

ACC: acetyl-CoA carboxylase
ALP: alkaline phosphatase
ALT: alanine aminotransferase
AMPK: AMP-activated protein kinase
apoB100: apolipoprotein B100
ARB: angiotensin receptor blocker
AST: aspartate aminotransferase
CCL3: chemokine (C-C motif) ligand 3
CCL5: chemokine (C-C motif) ligand 5
CCR1: C-C chemokine receptor type 1
CCR5: C-C chemokine receptor type 5
CD36: cluster of differentiation 36
ChREBP: carbohydrate regulatory element-binding protein
CPT1: carnitine palmitoyltransferase 1
DGAT2: diacylglycerol O-acetyltransferase 2
DPP-4: dipeptidyl peptidase-4
ER: endoplasmic reticulum
FABP1: fatty acid-binding protein 1
FAS: fatty acid synthase
FATP: fatty acid transport proteins
fCK18: caspase-generated fragmented cytokeratin 18
FDA: Food and Drug Administration
FGF: fibroblast growth factor
GGT: γ-glutamyl tranferase
GLP-1: glucagon-like peptide-1
GSK3β: glycogen synthase kinase 3β
HCC: hepatocellular carcinoma
HSC: hepatic stellate cell
HTN: hypertension
IL-1β: interleukin 1β
IR: insulin resistance
MAFLD: metabolic-associated fatty liver disease
MCP1: monocyte chemoattractant protein-1
mTOR: mammalian target of rapamycin
MTTP: microsomal triglyceride transfer protein
NASH: non-alcoholic steatohepatitis
NLRP3: NLR family pyrin domain containing 3
PDE4: phosphodiesterase-4
PEMT: phosphatidylethanolamine *N*-methyltransferase
PPAR: peroxisome proliferator-activated receptor
RCTs: randomized controlled trial
SCD1: stearoyl-CoA desaturase-1
SGLT2: sodium-glucose cotransporter-2
shRNA: short hairpin RNA
SIRT1: Sirtuin 1
SREBP1c: sterol regulatory element-binding protein 1c
TGFβ: transforming growth factor-β
THR-β: thyroid hormone receptor-β
T2DM: Type 2 diabetes mellitus
VLDL: very low-density lipoprotein
